# Tracking of Systemic Lupus Erythematosus (SLE) Longitudinally Using Biosensor and Patient-Reported Data: A Report on the Fully Decentralized Mobile Study to Measure and Predict Lupus Disease Activity Using Digital Signals—The OASIS Study

**DOI:** 10.3390/biotech12040062

**Published:** 2023-11-09

**Authors:** Eldon R. Jupe, Gerald H. Lushington, Mohan Purushothaman, Fabricio Pautasso, Georg Armstrong, Arif Sorathia, Jessica Crawley, Vijay R. Nadipelli, Bernard Rubin, Ryan Newhardt, Melissa E. Munroe, Brett Adelman

**Affiliations:** 1 Progentec Diagnostics, Inc., Oklahoma City, OK 73104, USA; glushington@progentec.com (G.H.L.); mpurushothaman@progentec.com (M.P.); fpautasso@progentec.com (F.P.); garmstrong@progentec.com (G.A.); asorathia@progentec.com (A.S.); jcrawley@progentec.com (J.C.); rnewhardt@progentec.com (R.N.); mmunroe@progentec.com (M.E.M.); badelman@progentec.com (B.A.); 2 GSK, Philadelphia, PA 19104, USA; vijay.r.nadipelli@gsk.com; 3 GSK, Raleigh, NC 27709, USA; bernard.x.rubin@gsk.com

**Keywords:** SLE, digital, biosensor, patient-reported outcomes, signs and symptoms of flare, real-world evidence

## Abstract

(1) Objective: Systemic lupus erythematosus (SLE) is a complex disease involving immune dysregulation, episodic flares, and poor quality of life (QOL). For a decentralized digital study of SLE patients, machine learning was used to assess patient-reported outcomes (PROs), QOL, and biometric data for predicting possible disease flares. (2) Methods: Participants were recruited from the LupusCorner online community. Adults self-reporting an SLE diagnosis were consented and given a mobile application to record patient profile (PP), PRO, and QOL metrics, and enlisted participants received smartwatches for digital biometric monitoring. The resulting data were profiled using feature selection and classification algorithms. (3) Results: 550 participants completed digital surveys, 144 (26%) agreed to wear smartwatches, and medical records (MRs) were obtained for 68. Mining of PP, PRO, QOL, and biometric data yielded a 26-feature model for classifying participants according to MR-identified disease flare risk. ROC curves significantly distinguished true from false positives (ten-fold cross-validation: *p* < 0.00023; five-fold: *p* < 0.00022). A 25-feature Bayesian model enabled time-variant prediction of participant-reported possible flares (P(true) > 0.85, *p* < 0.001; P(nonflare) > 0.83, *p* < 0.0001). (4) Conclusions: Regular profiling of patient well-being and biometric activity may support proactive screening for circumstances warranting clinical assessment.

## 1. Introduction

Chronic autoimmune conditions like systemic lupus erythematosus (SLE) create significant challenges for both healthcare systems and patients. This is because they often require frequent and unpredictable medical attention due to varying levels of disease activity and volatility of clinical disease flares [1]. SLE is a complex and devastating disease whose prevalence ranges from 73 to 100 cases per 100,000 reported from population-based studies [1,2,3,4] up to as high as 406 per 100,000 in African American women [5]. Disease onset often occurs in young adults, particularly in women of childbearing age and minorities. Given that disease onset often occurs in young adults, many SLE patients require consistent medical care for decades of their lives. Morbidity and early mortality associated with permanent organ damage driven by the frequency and severity of disease flares thus lead to significant direct and indirect medical costs [1] with the estimated annual economic burden at USD 31 billion. The annual treatment costs for patients suffering clinically diagnosed severe SLE flares has been estimated to be as high as USD 49,754 per patient with inpatient, outpatient, and pharmacy costs included [6].

The clinical manifestations of SLE are highly heterogenous and patients are classified, rather than diagnosed, as no definitive diagnostic test for disease classification exists [7,8,9]. SLE disease activity varies significantly across individuals but on average each patient is at risk of experiencing 1.8 flares per year. Clinical disease flares as well as the medications used to treat flares, including steroids, increase the risk of permanent organ damage, morbidity, and early mortality [10]. The way we currently treat and manage SLE is mostly responsive. This is because the tools we have for assessing clinical disease activity and flares, such as validated clinical instruments [11,12,13,14,15,16,17] that include the Safety of Estrogens in Lupus Erythematosus National Assessment-Systemic Lupus Erythematosus Disease Activity Index (SELENA-SLEDAI) for disease activity [13] and flare [18], as well as lab tests [19,20] do not predict future symptoms that might need medical attention. Due to the present and anticipated shortage of rheumatologists trained in using these validated clinical tools, along with the time they require and their subjectivity, there is a growing need for new methods of clinical assessment. Ideally, these new approaches should predict the risk of future heightened clinical disease activity and flares.

The identification of biometric factors to reliably predict the probable risk of increased clinical disease activity and/or flare is essential to enable proactive intervention to prevent or reduce the severity of clinical disease flares and the resulting short- and long-term organ damage. Intermittent clinical visits (every 3–6 months) for clinical evaluation and laboratory testing alone do not accurately capture the impact of physiological, environmental, and psychosocial factors related to the complexities of SLE. For several chronic diseases, high-resolution digital data from wearable devices interfaced with smartphone apps have been utilized to quantify physiological activities such as physical activity (walking/exercise), sleep duration and depth, heart rate variability, memory, and voice pattern variation [21,22,23,24,25,26,27]. Several published papers have addressed the validity of the Withings smartwatch device used in this study [28,29,30,31]. Patients also use apps to complete periodic surveys to collect PROs and real-world evidence (RWE). Mounting evidence continues to support the utility of wearables interfaced with mobile phone apps for monitoring symptoms and promoting positive self-management behaviors in chronic disease management.

The effectiveness of remote monitoring by mobile phone was originally demonstrated for several chronic diseases such as diabetes [21,22], cardiovascular disease [23,24], SLE, [25], Parkinson’s disease [26], and rheumatoid arthritis (RA) [27]. In a study of 82 RA and 73 axial spondyloarthritis (axSpA) patients, physical activity was measured by activity trackers, and the output was analyzed by machine learning. Patient-reported symptoms of possible disease flares were associated with less physical activity in both RA and axSpA [27]. Application of machine learning showed that changes in physical activity patterns were associated with patient-reported symptoms of possible disease flares with 96% sensitivity and 97% specificity. Clearly, the opportunity exists for machine learning models derived from wearable physiological trackers and PROs to enable real-time active disease monitoring.

The collection of digital data from connected wearable devices and smartphone apps offers a viable avenue in SLE to explore the complex and dynamic nature of clinical disease phenotypes in real time. This fully decentralized longitudinal, observational study used the LupusCorner Research App (LCR-App) interfaced with the Withings Steel HR to collect biosensor-detected physiological digital signals (e.g., activity level, sleep duration and depth, heart rate variability) and complete PRO survey data. We determine the potential of this data to predict changes in self-reported symptoms of SLE disease activity and possible clinical disease flares that warrant further clinical evaluation.

## 2. Materials and Methods

### 2.1. Study Design

OASIS was conducted as a fully decentralized study. The overall study design was informed by survey data and overall web/app traffic analyzed from the LupusCorner community of 120,000 patients and their caregivers. Additionally, the LupusCorner Patient Innovation Council provided feedback on a range of protocol decisions including wearable device options, survey questions, and the frequency of events.

### 2.2. Recruitment

A study landing page was created, and a combination of digital marketing strategies was used to recruit an interested and appropriate audience to LupusCorner. A Facebook group of the same name with ~36,000 followers was also utilized to publicize the study. Interested potential participants signed up by answering pre-screening questions on a web app. If the participant passed pre-screening criteria, they subsequently had to meet the following eligibility criteria: (a) female or male age 18 or older; (b) currently not enrolled in another study; (c) able to understand the requirements of the study, provide written informed consent, including consent for the use and disclosure of research-related health information, and comply with the study data collection procedures. Participants were excluded for the following reasons: (a) under 18 years old; (b) known to be pregnant; (c) not able to understand the requirements of the study, provide written informed consent, including consent for the use and disclosure of research-related health information, or comply with the study data collection procedures.

### 2.3. Consent

Participants meeting screening and eligibility criteria interested in participating in the study electronically reviewed and signed the WCG-IRB-approved study consent form. Each participant was asked if they agreed to participate and acknowledged that they had read and understood the consent form, as well as confirming that they were aware of how to contact the study coordinator if they had any unanswered questions. Following completion of the electronic consent form, each participant was supplied an electronic copy of the signed form via e-mail.

### 2.4. LCR-App

The LCR-App is our HIPAA and CFR 21, Part 11-compliant platform for collecting and processing research-grade digital signals, as well as survey and medication data from SLE patients. The LCR-App was developed using the FDA MyStudies open-source codebase to create a custom, branded implementation of this platform [32]. The original codebase was developed by the FDA to ensure data integrity and security when conducting digital research and clinical trials [32]. The LCR-App is available for both iOS and Android and enables the collection of patient-reported outcomes (PROs) and the gathering of real-world evidence (RWE) data. The LCR-App supports fully remote studies including digital enrollment and administration of informed eConsent. The LCR-App also has the capability to use on-phone sensors and features, such as the camera, to record biometrics and supports the integration of commercial devices. In this study, the LCR-App was integrated with a Withings wearable (discussed in detail below).

### 2.5. Study Tasks

**Figure 1:**
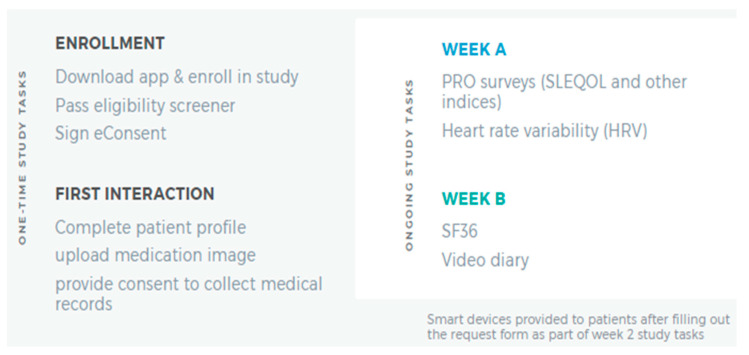
Summary of Initial Study Tasks. The LCR-App was downloaded, and subsequent study tasks completed in the app.

Participants completing the consenting process were given the LCR-App. Figure 1 summarizes one-time and ongoing study tasks the participants completed. Using the app, the participants completed a 20 min survey and 4 activities within 7 days of enrolling in the study. In total, this one-time assessment which involved completing the following five tasks: Patient Profile, Phone Verification, Location Sharing, Friend Referral, and Prescription Image Capture (Figure 1, left panel) was estimated to take less than 30 min. Following completion of these tasks, participants completed two 15 min surveys, biweekly, in the LCR-App concerning how their SLE, or general wellness, had affected their daily life in the past two weeks.

On a semi-weekly basis, the participants used their phone device camera to calculate Heart Rate Variability by placing a finger on the device camera for two minutes. In weeks where they did not complete the Heart Rate Variability test, participants were prompted to provide a brief, optional (approximately one to two minute) ‘selfie’ video on how their life was impacted by SLE in the last seven days (Figure 1, right panel).

### 2.6. Wearable Device

All active survey participants were offered the opportunity to receive a Withings Steel HR smartwatch [33] free of charge. A total of 144 out of 550 participants (26%) consented to wearing a smartwatch. Following the introductory survey, study qualification, and the completion of the first non-introductory weekly assignment, interested participants were shipped the smartwatch. If requested, our clinical coordinator assisted the participant in downloading the LCR-App and pairing the smartwatch with the app.

**Figure 2:**
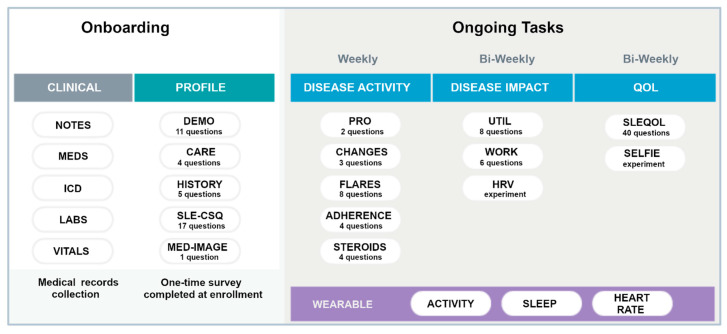
Summary of the onboarding and ongoing tasks in the full study. Different PROs are collected either weekly or Bi-weekly. The wearable watch was to be worn continuously. Definition of terms: MEDS—medications; ICD—ICD-10 codes; SLE-CSQ—connective tissue disease screening questionnaire; MED IMAGE—medical imagining; UTIL—resource utilization; HRV—heart rate variability; SLEQOL—SLE quality of life.

The smartwatch collects user activity in fivemin increments through accelerometers [27,33,34] and is Bluetooth equipped to communicate with most modern smartphones. It automatically measures and records physical activity (steps/distance), heart rate, and sleep duration/quality. It has a long-life battery that only requires recharging every 28 days and is water resistant up to 50 m. Data collected by the watch is transferred to the paired iOS/Android smartphone through the accompanying Health Mate app, which offers an API function to exchange user’s collected data to the LCR-App. Figure 2 summarizes the onboarding tasks and the frequency of data collection for ongoing tasks for participants wearing the smartwatch. Specific PROs were collected either weekly or bi-weekly as shown. The SLE Quality of Life Questionnaire (SLEQOL) was utilized to evaluate the participant’s disease-specific quality of life [35]. This SLE-specific standardized survey has been widely utilized and validated against other more generic quality of life surveys [36]. Participants were asked to wear the smartwatch both day and night, keep the battery charged as required, and allow access to the collected data in the LCR-App.

### 2.7. Participant Medical Records Data Processing

Medical records were obtained and analyzed for 68 individual participants in the study who were regular participants in OASIS data collection for at least 90 days. All medical records were subjected to optical character recognition text keyword extraction via the VietOCR program [37].

From these records, a visual inspection was made for a sample of six participants (all represented by different medical providers) to identify keywords of plausible SLE relevance that were recorded in a textually consistent manner and were tracked for more than one of the six participants examined. All resulting search terms, the associated medical concept, and the incidence (total number of detected instances, plus the number of patients with at least one mention) were recorded and transcribed.

### 2.8. OASIS Participant Stratification Based on Medical Records

All medical record terms relating to the concept of ‘flare’, flares’, or ‘flaring’ were automatically detected, counted, and manually scored as one (1) if the instance seemed to reflect a direct observation or account of either a possible SLE flare or a common co-pathology, one-half (0.5) if an unconfirmed implication that a flare might be occurring or have occurred recently, or zero (0) if either a negative finding or a purely incidental mention. From this analysis, an ordinate value for patient/participant possible flare vulnerability was derived as a fraction of the total manual score divided by the total automated count. These values were then converted into distinct qualitative classes by defining patients with scores of less than 0.4 as having low possible flare-risk, those with scores greater than 0.6 as having high possible flare risk, and those with intermediate value (between 0.4 and 0.6) as being of intermediate or ambiguous possible flare risk.

The independent variables entail a non-uniform number of time-varying OASIS observations collected for each study participant. Given the prospective information value contained in the time variation of these *x_i_* variables, the full OASIS dataset was recast as a superset of the mean (x), variance (σ^2^), maximum (x_max_), and range (x_max_–x_min_) of each quantity. This large set of metrics was then objectively parsed using a diverse range of feature selection techniques available in Weka [38] which, through experimentation, led to the conclusion that a 10-parameter subset of variables derived using the Gain Ratio Attribute algorithm fostered significant discrimination between patients with strong flaring risk versus those with little or no risk, while a semi-distinct 20-parameter set derived from the ReliefF algorithm produced much better delineation of those patients with marginal vulnerability. A summation set of 24 variables was aggregated from the 2 descriptor sets to craft a composite descriptor basis sensitive to both the factors that distinguish high and low possible flare vulnerability and those that characterize patients with intermediate risk. Diverse classification algorithms (also available in Weka) were then explored to identify schemes that produced strong levels of possible flare-risk delineation.

### 2.9. Analysis

Summary statistics were used to describe patient demographic and clinical characteristics of the study population with descriptive statistical analyses conducted where appropriate to determine mean, standard deviation, median, maximum, minimum, 95% confidence intervals, and *p*-values, all computed in Google Sheets using built-in functions for accuracy estimate for a single-variable expectation value (*x_i_*) or for a single difference (*x_i_*–*x_j_*) resulting from two-variable comparison for locally normal distributions [39]. The frequency and percentages of categorical metrics were determined from consideration of explicit data.

### 2.10. Data Processing and Mining of Survey and Biometric Data

Artificial intelligence (AI) provides a wealth of tools available to perceive operationally practical relationships in complex data, and numerous applications in healthcare are emerging [40,41]. Drawing on analytical capacities far greater than conventional statistics, AI enables the assimilation of disparate data types toward the prediction of loosely associated phenomena. In patient health assessment, AI can conceivably enhance pathology monitoring strategies or refine treatment schemes based on disparate information such as patient profiles, periodic self-assessments of life quality and disease activity, and biometric measurements.

In seeking to assimilate such disparate data, a key challenge entailed accommodating substantial variations in density and regularity of different recorded variables, as arose from imperfect compliance by participants in adhering to information collection protocols. To address such variations, a variety of non-linear functions have been explored, which tend to rely on numerically populating sparse regions between explicit reports [42,43,44,45,46]. To this end, we implemented a paradigm that first entails the elimination of each data point that is temporally isolated (i.e., for which there are no comparable data points available within the two weeks immediately before or immediately after the point in question), then de-prioritized all data corresponding to participants who did not sustain activity in the study for at least three months (i.e., data for short-term/temporary participants is not utilized for complex modeling). As is common practice in AI treatment of data with non-homogeneous density, gaps within this reduced dataset were then closed by linear interpolations between temporally proximal measurements [42].

The resulting data, spanning more than 200 parameters with potential influence on several dozen pathological metrics (and with further possible extensions to terms mined from medical records), were applied to inquiries regarding possible relationships between specific biometric and life-quality observations versus tangible health and pathology outcomes. To illustrate how medical observations might relate to the level of patients/participants, the medical record texts and the OASIS metrics were subjected to parsing and reconditioning.

## 3. Results

### 3.1. Participant Demographics

A total of 550 participants (self-reported SLE patients) consented and actively participated in the study. The majority (96%) of the participants were female, with an average age of 44 (SD ± 14) years (Table 1). The high percentage of female participants is expected because SLE is nine times more frequent in women than men. The self-reported racial/ethnic demographics for all participants and the 68 participants used in the modeling are shown in Table 1. Most participants reported being of European American (74.4%) descent followed by African American (10.4%) and Hispanic ethnicity (5.6%) while the distribution of the medical record subset was 85.3% European American with African Americans and Hispanics at 5.9% and Native Americans at 2.9%.

Most participants (86%) reported receiving their SLE diagnosis from a rheumatologist, 9% reported diagnosis by a family practice doctor, and 5% reported diagnosis by “other doctor.” Similarly, most participants (87%) reported receiving their SLE care from a rheumatologist, 9% reported receiving care from a family practice doctor, 1% reported “other doctor” and 3% reported receiving no medical care.

### 3.2. Patient-Reported Medication Usage

**Figure 3:**
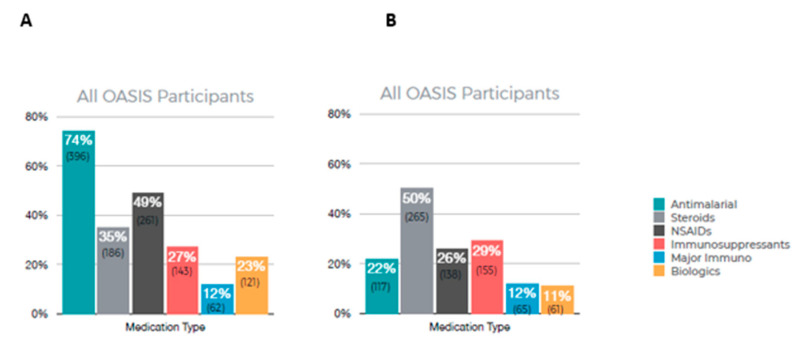
Participant-reported medication usage. (**A**) Current medication being used, (**B**) medication reported no longer taking.

Figure 3 summarizes the results of patient-reported medications currently in use (A) and medications that are no longer in use (B). Among all OASIS participants, the most frequently used medication was antimalarials (74%) with 49% reporting using NSAIDs. A total of 50% of participants reported no longer using steroids and 29% reported no longer using immunosuppressants.

### 3.3. Medical Records Data

Supplementary Table S1 shows the medically relevant keywords identified from medical records whose presence could be productively quantified and used as search terms as described in the methods. Also presented are the associated medical concept and the incidence (total number of detected instances, plus the number of patients with at least one mention) identified in the medical records of the sixty-eight long-duration OASIS participants.

**Figure 4:**
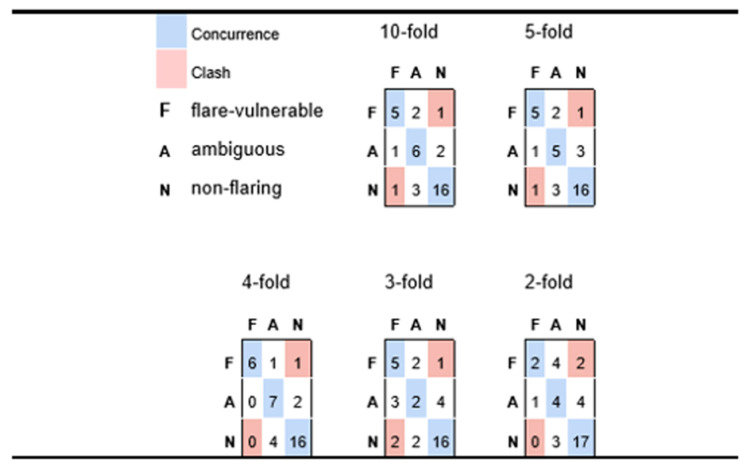
Confusion matrices for participants with possible flare history (F), versus ambiguous (A) and likely non-flare (N), predicted via Naïve Bayes for varying levels of cross-validation. Each row corresponds to a true medical class and reports how participants in that class are classified, whereas columns correspond to how class members are sorted into predicted classes. Diagonal cells reflect accurate predictions; off-diagonals are discordances.

The outcomes of applying diverse classification algorithms to identify schemes that produced strong levels of possible flare-risk delineation are shown in Table 2 and Figure 4. From Table 2 we note that the final models typically achieve strong delineation of possible flare-resistant patients (both in terms of precision and recall), while recall of possible flare-vulnerable patients is somewhat lagging, which implies that the model risk is missing some participants whose profile is indicative of a possible flare risk.

However, from the results in Figure 4 we conclude that most false negative predictions for possible flare-risk patients fall into the ‘ambiguous’ category which, from the perspective of practical medicine, would be grounds for cautioning a patient of a possible impending risk. Indeed, in Figure 4 we see very minimal instances of predictive disagreement, wherein an at-risk patient is predicted to incur minimal chance of possible flares, or a patient with no symptoms of possible flare reports is nonetheless predicted to be at elevated risk.

Table 2 suggests that the model is quite robust because only a slight decline in precision and recall was observed when increasing the stringency of the cross-validation conditions from 10-fold to 5-fold. Figure 4 takes this assertion further, in showing predictive performance as one goes to even more stringent conditions inherent in four-fold, three-fold, and two-fold cross-validation. In fact, the very best aggregate predictivity is observed when applying four-fold cross-validation constraints, although it is reasonable to assume that this may be more accidental than fundamental (i.e., predictive performance is rarely better for models based on 75% of available data than those generated from a basis of 80%). Nonetheless, it is reasonable to assert good predictivity for the four-fold case, which implies a model of reasonable generality and extensibility. The three-fold experiment retains a fair capacity for distinguishing possible flare-susceptible patients from those with low risk, but less sensitivity for assessing patients with intermediate risk. Even the two-fold experiment appears to suggest some predictive value, especially given that the recorded predictions yield only two outright clashes (two participants who had elevated medical vulnerability to flaring but were predicted to be low risk); however, the small number of patients (three) that are predicted to be possible flare vulnerable suggests that the predictive framework is descending into a common pitfall for classification of unbalanced outcomes, whereby the model sorts a disproportionate number of instances into larger classes, thus depleting minority classes.

Beyond the capacity of a simple model to identify more possible flare-vulnerable participants from within the survey participants, there is an equally tangible value in identifying factors that lead to such differentiation. Our 24-metric descriptor set exhibited conceptual diversity, with key factors including terms like the average biometrically determined nocturnal wakefulness, variance in steroid usage, and average and maximal values of Raynaud’s symptoms like finger color. Several other symptom-related metrics were found to be important; however, the variances and ranges in those observations were generally more important than their means or maxima.

It is finally worth noting that multiple factors relating to interpersonal support networks and responsibilities influenced the predictions. One factor that has surfaced in multiple distinct models as an important metric for differentiating outcomes relates to whether the participant had childcare responsibilities (children, caretaker.value). Those participants responsible for the care of at least one other person were significantly more likely to experience medically assessed possible SLE flares.

Table 3 shows the relative predictive accuracy for self-reported symptoms of possible disease flares (TP-possible flare) and self-reported likely non-flares (TP-no) as a function of 7 different feature selection techniques (25 highest weighted features selected in all cases), in the context of directed graph (Bayes), deep neural network (multi-layer perceptron), and decision tree (LMT) methods. All prediction rates are derived from 10-fold cross-validation analysis.

Table 4 shows the relative capacity of Bayesian network models to accurately predict self-reported symptoms of possible disease flares (TP-flare) and self-reported likely non-flares (TP-no) as a function of three different feature sets, as validated by five different levels of cross-validation stringency.

This 25-feature model aimed at identifying time-variant circumstances predicting potentially medically relevant physical discomfort (as gauged by QOL symptoms of possible flare activity self-reports) yielded Bayesian networks that, over rigorous levels of cross-validation, achieved useful true-positive and true-negative predictive accuracy for symptoms of possible disease flare self-reports (P(true-possible flare) > 0.85; *p* < 0.001; P(true-likely non-flare) > 0.83; *p* < 0.0001). The most informative terms were biometric sleep data, the presence or absence of various epithelial lesions, and several QOL terms.

## 4. Discussion

Remote care of complex chronic or intermittent diseases such as SLE requires the effective monitoring of a reasonable array of readily measurable patient health and physical state parameters with some bearing on the disease state. Digital data from connected wearable devices and smartphone apps offer a novel avenue to explore the complex and dynamic nature of SLE clinical disease activity. Participants thus completed PRO surveys in the LCR-App and had digital signals of their daily activities collected by the same app interfaced with the wearable smartwatch. These non-medical metrics were augmented for a subset of patients via digitized medical records data. Medical records data were collected and digitized. Advanced data mining and machine learning were then applied to these data to develop and validate digital predictive models for symptoms of SLE disease activity that may indicate a clinical flare and require additional clinical assessment. Machine learning, a subset of AI, includes algorithms to enable computers to detect patterns from large complex datasets, learn important features, and use these patterns to make predictions about previously unseen data. Digital phenotyping, defined as the “moment-by-moment quantification of the individual-level human phenotype in situ using data from personal digital devices” has emerged as a viable method to conceptualize such data to improve health [47].

Regular profiling of patient self-reported well-being and biometric activity offers promising screening potential to identify patients in need of additional clinical assessment. Our data analysis determined that potentially predictive relationships are derivable for anticipating specific patient metrics that predispose patients to suffering symptoms of a possible SLE flare (Table 2). Specifically, five-fold and ten-fold cross-validated models aimed at predicting flare severity yield statistically significant results. These models provide insight into the prospective predictive capacity derived from aspects of patient demographics, medical histories, biometric data, and self-reported QOL metrics.

A limitation of the current study arose from the fact that much of the data directly relating to the characterization of the SLE pathology state was self-reported, which is not guaranteed to align with the rigorous pathological assessments conducted by trained medical professionals. For a subset of 68 participants, medical records were provided in conjunction with participant visits to, and communications with, care facilities and medical personnel. Unfortunately, these interactions occurred sporadically, without controlled correspondence to regularly collected QOL and biometric data. In addition, while each record is dated, many records contain non-specific observations that lack temporal precision. For example, there are frequent instances of non-specific phrases such as “the patient has been experiencing…”, without specific time-stamp metadata that could permit causality inferences within the context of the regularly collected QOL and biometric data. Encoding temporal causation (i.e., prediction of observables such as self-reported symptoms of possible disease flares) poses challenges because of data sparsity such as variables that are not measured every day, and concurrent measurements of independent variables (e.g., survey inputs or biometric records) are frequently missing for days on which dependent variables (i.e., symptoms of possible disease flare self-reports) are recorded. Finally, many participants displayed greater dedication to one aspect of data collection (i.e., surveys vs. biometrics) while letting other aspects lapse. On a practical level, the result of inconsistent participation is sparse, non-uniform data wherein factors that might influence disease activity, or reflect favorable treatment profiles, are not guaranteed to be reported in close temporal alignment with those trends.

A second factor that extenuates the concern of data sparsity is the relatively small sample size which, in the perspective of the relative heterogeneity of SLE, produces a scenario wherein specific relationships between observable metrics and disease circumstances may not be fully robust (i.e., suggestive trends may actually be statistical accidents). In this light, it is important to view this study as a preliminary basis for constructing longer-term longitudinal studies that can better validate proposed relationships, as well as expose novel dependencies that were not evinced from this initial cohort. As preparation for such refinements, our initial exploration deliberately introduced a metric basis that covered a diverse range of different prospective data types and sources. Refined studies will likely lead to judicious triage of some preliminary metrics but will be better positioned to avoid the ab initio omission of important considerations.

Fortunately, to a reasonable extent, artificial intelligence may circumvent deficiencies such as data sparsity through various schemes such as interpolative methods that determine how independent measurements at points proximal to the predicted observable event may influence the dependent observation. While a variety of non-linear functions have been explored for populating sparse regions between explicit reports [33,34,35,36,37], we used simple linear extrapolation to estimate the values of independent parameters at the time of measurement of the predictable observable, based on any records of independent variables within (but not exceeding) two weeks of the dependent observation.

To enhance the medical diagnostic rigor of the OASIS model, a fuller and more comprehensive data collection effort will be required to bridge areas of sparseness, and to contextualize specific medical interventions rigorously and temporally within the QOL and biometric input flow. Indeed, the collection of participant medical records is ongoing with the goal of adding more clinical data into this unique digital dataset to further develop, refine, and validate both the protocols for information-rich data acquisition and the enhanced predictive models that may arise. In particular, in the future, the team aims to replace the static QOL survey with an adaptive, generative conversational interface [48]. Tailored to employ medically effective communication strategies such as those encoded within Swanson’s Theory of Caring [49], the interface will procure more accurate and temporally fine-grained information from participants, with three overarching synergistic goals: (a) to better understand patient-specific metrics that may relate to transitions between stages of health and pathology; (b) to compile trends across the full participant cohort to identify cross-cutting metrics from (a) that appear in a significant fraction of cases, thus potentially demarcating a diagnostically relevant subset, and (c) to use information from (a) and (b) to devise ‘trigger’ conditions under which the input acquired from a given participant at a given time appears to be sufficiently problematic as to prompt automated contact with a live medical professional to directly assess the participant.

Overall, the results from the current pilot study strongly suggest that regular profiling of self-reported well-being and biometric activity has the potential to identify patients at risk of symptoms of possible imminent increased disease activity and flare, thus indicating the necessity for clinical assessment and/or intervention. In conjunction with a more adaptive methodology for acquiring reliable and temporally interpretable medical assessments with which to better train and validate our predictive models, we are confident that this research will lay the groundwork for a shift in SLE management paradigms in clinical practice, whereby clinical phenotypes may be derived from digital signals collected by mobile apps and wearable sensors. This novel, digital biomarker platform not only captures data but may also offer impactful information for improving patient health and quality of life.

## Figures and Tables

**Table 1:** Demographic features of the OASIS study participants (n = 550) and MR subset (n = 68).

Sex (n, %)	Female Male	529 (96) 21(4)	68 (100) 0 (0)
Race/Ethnicity (n,%)	European American	409 (74.4)	58 (85.3)
	African American	57 (10.4)	4 (5.9)
	Hispanic	31 (5.6)	4 (5.9)
	Asian	25 (4.5)	0 (0)
	Other	12 (2.2)	0 (0)
	Native American	11 (2.0)	2 (2.9)
	Unknown	5 (0.9)	0 (0)
Age (Mean, SD)		44 (14)	44 (13)

**Table 2:** Performance statistics of Naïve Bayes classification of possible flare-vulnerable vs. ambiguous vs. non-flaring patients, as predicted from OASIS metrics, at the levels of 10-fold and 5-fold cross-validation.

10-Fold CV	TP	FP	Precision	Recall	F-Measure	ROC	Class
Average	0.63	0.07	0.71	0.63	0.67	0.76	flares
	0.67	0.18	0.55	0.67	0.60	0.67	ambiguous
	0.80	0.18	0.84	0.80	0.82	0.74	non-flare
	0.73	0.15	0.74	0.73	0.73	0.73	
5-Fold CV	TP	FP	Precision	Recall	F-measure	ROC	Class
Average	0.63	0.07	0.71	0.63	0.67	0.76	flares
	0.56	0.18	0.50	0.56	0.53	0.56	ambiguous
	0.80	0.24	0.80	0.80	0.80	0.69	non-flare
	0.70	0.19	0.71	0.70	0.71	0.67	

The corresponding coding labels and metrics applied in this analysis are listed in the Supplementary Materials.

**Table 3:** Relative predictive accuracy for self-reported flares (TP-flare) and self-reported non-flares (TP-no) as a function of seven different feature selection techniques (25 highest weighted features selected in all cases), in the context of example graph (Bayes), deep neural (multi-layer perceptron) and decision tree (LMT) methods. All prediction rates are derived from 10-fold cross-validation analyses.

	Bayesian Network		Multilayer Perceptron		LMT	
	TP-flare	TP-no	TP-flare	TP-no	TP-flare	TP-no
Correlation Feature Subset (CFS)	0.907	0.864	0.611	0.928	0.574	0.953
Classified Attribute (ClA)	0.907	0.703	0.352	0.892	0.296	0.961
Correlation Attribute (CoA)	0.907	0.792	0.630	0.928	0.556	0.946
Gain Ratio Attribute (GRA)	0.944	0.742	0.593	0.950	0.500	0.943
Information Gain Attribute (IGA)	0.907	0.792	0.648	0.939	0.574	0.957
One R Attribute (ORA)	0.593	0.932	0.593	0.921	0.537	0.961
Relief F Attribute (RFA)	0.944	0.713	0.630	0.939	0.500	0.953
Symmetry Uncertain Attribute (SUA)	0.926	0.778	0.611	0.953	0.556	0.961

**Table 4:** Relative capacity of Bayesian Network model to accurately predict self-reported flares (TP-flare) and self-reported non-flare (TP-no) as a function of three different feature sets, as validated by five different levels of cross-validation stringency.

	Set 1: (CFS-25)		Set 2 = {Set 1 + 12 Biometric Terms}		Set 3 = {Set 2 Sub-Selected by Bayes-Steered CIA}	
	TP-flare	TP-no	TP-flare	TP-no	TP-flare	TP-no
10-fold	0.907	0.864	0.907	0.860	0.944	0.875
5-fold	0.889	0.885	0.889	0.889	0.944	0.878
4-fold	0.907	0.867	0.907	0.864	0.944	0.839
3-fold	0.852	0.878	0.870	0.878	0.926	0.860
2-fold	0.796	0.885	0.852	0.878	0.852	0.871

The corresponding coding labels and metrics applied in this analysis are listed in the Supplementary Materials.

## Data Availability

Data will be made available, as appropriate, under the guidelines of our IRB approvals for safeguarding the privacy and data of research participants and our duty to protect confidential and proprietary data and third-party intellectual property. Data sharing will be subject to agreement to Progentec Diagnostics’ Materials Transfer Agreement which specifies the way the data may be used, and by whom.

## Supplementary Materials

The following supporting information can be downloaded at https://www.mdpi.com/article/10.3390/biotech12040062/s1, Table S1: Text search term instances and frequencies in the medical records of 68 long-duration OASIS participants; Text S1: Corresponding coding labels and metrics for Table 2; Text S2: Corresponding coding labels and metrics for Table 4.
