# Supplementary material for: Tracking of Systemic Lupus Erythematosus (SLE) Longitudinally Using Biosensor and Patient-Reported Data: A Report on the Fully Decentralized Mobile Study to Measure and Predict Lupus Disease Activity Using Digital Signals—The OASIS Study
Source: BioTech (Basel). 2023 Nov 9;12(4):62. doi: 10.3390/biotech12040062 (PMC10660535; doi:10.3390/biotech12040062)
Supplement: Supplementary file 1 [file biotech-12-00062-s001.zip › biotech-2609976-supplementary.pdf]

**Supplementary Data**

**Table S1** shows the medically relevant keywords identified from medical records whose presence could be productively quantified and used as search terms as described in the methods. **Table S1** also shows the associated medical concept and the incidence (total number of detected instances, plus the number of patients with at least one mention) identified in the medical records of the 68 long-duration OASIS participants.

**Table S1. Text search term instances and frequencies in the medical records of 68 long-duration OASIS participants**

| Search Term | Target Concept | Total Hits | Unique Patients | Search Term | Target Concept | Total Hits | Unique Patients |
|---|---|---|---|---|---|---|---|
| adenopath | adenopathy/ic | 394 | 42 | leukocyt | leukocyte | 704 | 34 |
| albumin | albumin | 806 | 49 | lump | lump | 54 | 17 |
| allerg | allergy/gies/gic | 1991 | 63 | lymph | lymph (multi vars) | 1871 | 57 |
| anemi | anemia/c | 213 | 31 | lymphocyt | lymphocyte/tic | 650 | 38 |
| anxi | anxious/anxiety | 488 | 41 | memory | memory | 58 | 23 |
| appetite | appetite | 116 | 23 | monocyt | monocyte/tic | 646 | 40 |
| thralgi | arthralgia | 540 | 39 | murmur | murmur | 271 | 39 |
| auscult | auscultation | 378 | 41 | yalgi | myalgia | 924 | 41 |
| axillary | axillary | 229 | 17 | naproxen | naproxen | 41 | 9 |
| glycoprotein | (b2/Beta 2)-glycoprotein | 165 | 22 | nause | nausea/nauseous | 563 | 54 |
| basophil | Basophil | 566 | 35 | neurolog | neurologic/al | 645 | 47 |
| benlysta | Benlysta | 1283 | 33 | neuropath | neuropathy/ology | 161 | 14 |
| bilirubin | Bilirubin | 733 | 48 | neutroph | neutrophil | 825 | 45 |
| bleed | bleeds/bleeding | 180 | 39 | sweat | night sweats | 161 | 35 |

| | | | | | | | |
|---|---|---|---|---|---|---|---|
| bruis | bruise/bruising | 234 | 38 | nitrite | nitrite | 344 | 42 |
| C-reactive | C-reactive protein | 644 | 55 | nodul | nodule | 309 | 29 |
| cardiolipin | Cardiolipin | 273 | 28 | nsaid | NSAIDs | 144 | 28 |
| cholesterol | Cholesterol | 237 | 19 | numbn | numbness | 317 | 36 |
| cognit | cognitive/ion | 82 | 17 | pharyn | oro/pharynx/geal | 248 | 33 |
| olchicin | Colchicine | 140 | 12 | palpita | palpitation | 279 | 38 |
| complement | complement (multi vars) | 1847 | 53 | paraly | paralysis/paralyzed | 16 | 5 |
| congest | congestion/congestive | 112 | 19 | ericar | Pericarditis | 632 | 23 |
| conjunctiv | conjunctiva/itis | 357 | 46 | phosphatase | phosphatase | 382 | 40 |
| constipa | constipation/ted | 213 | 29 | platelet | platelet | 792 | 52 |
| cough | Cough | 464 | 47 | pleurit | pleuritic/is | 214 | 22 |
| creatinine | Creatinine | 1835 | 60 | nison | Prednisone | 1397 | 43 |
| depress | depressed/ion | 871 | 42 | psychiatr | psychiatric | 290 | 38 |
| diarrhea | Diarrhea | 425 | 44 | pulmon | pulmonary | 491 | 26 |
| dizz | dizzy/dizziness | 220 | 36 | rash | rash | 1903 | 62 |
| drain | Drainage | 56 | 16 | aynau | Raynaud's | 756 | 42 |
| ysphagi | Dysphagia | 90 | 17 | serosit | serositis | 38 | 9 |
| dyspnea | Dyspnea | 164 | 25 | sicca | sicca | 208 | 19 |
| dysur | Dysuria | 114 | 28 | gren | Sjogren's | 987 | 44 |
| edema | Edema | 696 | 49 | sleep | sleep/ing/iness | 609 | 54 |

| | | | | | | | |
|---|---|---|---|---|---|---|---|
| eosinophil | Eosinophil | 492 | 35 | spleen | spleen | 39 | 12 |
| fatigu | Fatigue | 1270 | 58 | squamous | squamous | 146 | 19 |
| ferritin | Ferritin | 89 | 13 | steroi | steroids | 333 | 39 |
| fever | Fever | 1009 | 56 | stiff | stiff/stiffness | 542 | 50 |
| finger | finger (multi vars) | 454 | 50 | swell | swell/swelling | 1669 | 57 |
| flar | flare/flaring | 558 | 55 | swoll | swollen | 272 | 38 |
| genitour | Genitouria | 159 | 26 | synovi | synovitis | 401 | 43 |
| GERD | GERD | 147 | 15 | achycardi | tachycardia | 57 | 8 |
| globulin | Globulin | 646 | 37 | thrombo | thrombosis/cytopenia | 145 | 20 |
| glucose | Glucose | 953 | 56 | thyroid | thyroid | 574 | 47 |
| hair | hair/hairline | 418 | 46 | tingl | tingling/tingle | 245 | 36 |
| headache | Headache | 544 | 49 | tobacco | tobacco | 832 | 46 |
| heartburn | Heartburn | 138 | 28 | tonsil | tonsil/lectomy | 51 | 13 |
| hematoc | hematocrit/chezia | 442 | 43 | ulcer | ulcer | 824 | 52 |
| ematur | Hematuria | 155 | 31 | urinal | urinalysis | 784 | 57 |
| hepat | hepatic/it is | 492 | 41 | urinary | urinary (multi vars) | 308 | 41 |
| hives | Hives | 146 | 24 | corti | various corts | 129 | 27 |
| oxychlo | hydroxychloroquine | 1031 | 53 | cobalam | various vit-B | 97 | 10 |
| infect | infected/tion | 799 | 53 | viral | viral | 50 | 16 |
| inflamm | Inflamm | 660 | 48 | vision | vision | 344 | 40 |
| intolera | intolerant/ce | 166 | 24 | vomit | vomiting | 304 | 42 |

| | | | | | | | |
|---|---|---|---|---|---|---|---|
| joint | Joint | 2330 | 61 | weakness | weakness | 285 | 45 |
| ketone | Ketone | 355 | 43 | weight | weight (gain/loss) | 1314 | 60 |
| lesio | Lesion | 395 | 42 | wheez | wheeze/wheezing | 366 | 38 |

*The corresponding coding labels and metrics applied in the analysis presented in Table 2.*

**Corresponding metrics:** d109a/wakeupduration (mean biometric length of nocturnal awakening); d139a/q16_fingers_color (mean frequency of temperature dependent finger-color change); d196a/q15_fingers_cold_sensitive (mean frequency for unusual cold-sensitivity in fingers); d046v/o5_hc_team (variance in whether outreach was sought to healthcare team); d048v/f1_symptoms (variance in whether symptoms are being reported); d056v/f2_nose_ulcers (variance in values relating to whether nose ulcers are being reported); d061v/f3_symptoms_duration (variance in the duration of reported symptoms); d062v/f4_med_assist(variance in whether medical assistance was sought); d067v/f6_contact_dr (variance in whether doctor was contacted); d077v/ma4_felt_better (variance in stopping medication due to feeling better); d079v/ma4_other (variance in stopping medication due to some other reason); d080v/s1_steroids (variance in response of whether currently using steroids); d081v/s2_number_days_steroids (variance for number of days on steroids); d046s/o5_hc_team (spread in reports regarding whether outreach was sought to healthcare team); d048s/f1_symptoms (spread in values relating to whether symptoms are being reported); d056s/f2_swollen_joints (spread in values relating to whether swollen joints are being reported); d062s/f4_med_assist (spread in whether medical assistance was sought); d067s/f6_contact_dr (spread in instances where doctor was contacted); d044m/o5_sig_other (maximum frequency in whether support/help was sought from significant other); d046m/o5_friend (maximum frequency in whether support/help was sought from friend); d062m/f3_symptoms_duration (maximum symptom duration reported); d067m/f6_change_meds (maximum in number of instances of changing medications); d139m/q16_fingers_color (maximum frequency of temperature dependent finger-color change); d236m/children. caretaker value (is the patient/participant responsible for childcare?)

*The corresponding coding labels and metrics applied in the analysis presented in Table 4.*

**Corresponding metrics:** d53/f2_sores (whether experienced lesions); d26/q26_med_inconvenience (whether experienced lifestyle inconveniences due to medical issues); d60/f2_other (whether experienced some detectable medical symptom other than those listed); d55/f2_nose_ulcers (whether nose ulcers are being reported); d37/q37_med_concern (impacts arisen that rise to medical concern levels?); d189/Sex (female or male); d54/f2_mouth_ulcers (whether mouth ulcers are being reported); d49/f1_symptoms (whether experienced some detectable medical symptom); d58/f2_depression (whether experienced psychological depression); d51/f2_rash (whether experienced a skin rash); d47/o5_other (whether support/help was sought from someone other than those listed); d72/f7_severity_to_past (how do symptom severities compare to past instances?); d8/q8_career_interferance (have medical circumstances impinged on career?); d6/q6_walking_3km (impacts on physical exercise?); d16/q16_memory (impacts on physical cognition/memory?); d42/o5_No (whether no support/help was sought from anyone); d17/q17_appetite (experienced a change in appetite?); d68/f6_visit_er (made any recent visits to ER?); d88/s4_mood_change (whether experienced detectable mood change(s)); d187/q37_other (impacts other than those listed?); d89/s4_insomnia (whether experienced insomnia); d105/durationtosleep (biometric duration in falling asleep); d106/durationtowake (biometric duration in awakening); d107/lightsleepduration (biometric duration of light sleep); d108/wakeupcount (biometric counts of nocturnal awakenings)
